# A selection index with minimal genetic relatedness for multi-trait data via binary quadratic programming

**DOI:** 10.1186/s13007-025-01484-4

**Published:** 2025-12-29

**Authors:** Osval A. Montesinos-López, Abelardo Montesinos-López, Carlos M. Hernández-Suárez, Admas Alemu

**Affiliations:** 1 Facultad de Telemática, Universidad de Colima, Colima, Colima 28040 Mexico; 2 Centro Universitario de Ciencias Exactas e Ingenierías (CUCEI), Universidad de Guadalajara, Guadalajara, Jalisco 44430 Mexico; 3 Coordinación General de Investigación Científica, Universidad de Colima, Colima, Colima 28040 Mexico; 4 Department of Plant Breeding, Swedish University of Agricultural Sciences, P.O. Box 101, Alnarp, 230 53 Sweden

**Keywords:** Plant breeding, Linear programming, Quadratic programing, Multi-trait index selection, Genetic diversity, Candidates individuals

## Abstract

Genomic selection (GS) in plant breeding aims to identify individuals with superior genetic merit while maintaining genetic diversity within populations. In plant breeding, considering multiple traits simultaneously makes optimizing selection complex, especially under genetic relatedness constraints. In this study, we propose a binary quadratic programming framework for constructing a multi-trait selection index that maximizes genetic gain while minimizing average pairwise relatedness appropriate for identifying superior candidates for advancement in the breeding pipeline. The approach combines estimated breeding values (EBVs) across multiple traits by applying trait-specific economic weights, while simultaneously accounting for coancestry through the genomic relationship matrix. By formulating the selection problem as a constrained Quadratic Programing Multi-trait Selection Index (QPMSI), our method enables the identification of a fixed number of candidate individuals that jointly optimize selection index values and control genetic relatedness. We evaluated the performance of the proposed method using five real genomic datasets and demonstrated that it provides a more effective balance between selection response and control of genetic relatedness than the Linear Programming Multi-trait Selection Index (LPMSI). In particular, the QPMSI consistently outperformed the LPMSI in terms of the MV metric (gain-to-degree of relatedness ratio), achieving improvements of at least 53.8%. This framework offers a practical and computationally efficient tool for sustainable breeding strategies in multi-trait selection contexts.

## Introduction

 One of the fundamental goals in plant breeding is to improve the genetic potential of crop species to meet global challenges related to food security, climate change, and sustainability. GS, first proposed by Meuwissen et al. [22], has revolutionized breeding by enabling the prediction of breeding values using dense genome-wide markers. Unlike the traditional marker-assisted selection that relies on significant associations, GS uses all available marker information simultaneously, allowing breeders to accelerate the selection cycle, enhance selection accuracy, and increase genetic gain per unit time [2, 8, 15]. Within the GS framework, one of the critical decisions breeders must consider is aggregating information from multiple traits and selecting the best individuals efficiently. This is where index selection becomes vital.

Index selection aims to identify superior genotypes by combining multiple traits of economic or agronomic importance into a single index. It enables simultaneous improvement of multiple traits, each potentially under different genetic architectures and economic weights [14]. The theoretical foundations of index selection date back to the early 20th century when Hazel introduced the concept of the linear selection index, which weighs traits based on their heritability, genetic correlations, and economic importance. Since then, index selection has become a cornerstone in animal and plant breeding programs, providing a formal strategy to balance trade-offs between traits and optimize overall genetic merit [3, 29].

In plant breeding, selection indices have traditionally been constructed using phenotypic and pedigree information. These indices are typically designed to maximize the expected genetic gain in a set of traits while maintaining selection on a manageable number of genotypes. Examples include tandem selection, independent culling levels, and the economic or restricted selection indices [10]. Among these, the economic selection index has been widely adopted due to its flexibility in incorporating economic weights and genetic covariances, making it suitable for improving complex traits such as yield, disease resistance, and quality simultaneously. However, these classical indices often rely on accurate estimation of genetic parameters and are limited by phenotyping costs and environmental variability.

The integration of index selection into the GS framework marks significant advancement [5]. With the availability of genomic estimated breeding values (GEBVs) for multiple traits, breeders can construct genomic selection indices (GSIs) that exploit marker-data information. This integration enhances the precision of multi-trait selection and allows for earlier selection of individuals, thereby accelerating genetic gain (Akdemir et al., 2016). Moreover, GS-based index selection facilitates the application of complex index models, such as restricted or desired-gains indices, without requiring extensive phenotypic data. Recent studies have demonstrated the successful use of GSIs in breeding programs for crops such as wheat [17], maize [19], and rice [27], showing notable improvements in selection response compared to univariate selection methods.

One notable benefit of applying index selection in GS is the ability to control traits that are difficult or expensive to phenotype but are genetically correlated with more easily measurable traits. This makes index selection particularly attractive in early-generation selection and in low-heritability trait scenarios. Additionally, breeders can incorporate sustainability-related goals, such as stress resilience or resource use efficiency, into the index without sacrificing productivity traits.

Despite these advances, several challenges remain in implementing GS-based selection indices effectively. A critical concern is the potential reduction of genetic diversity that accompanies intensive selection based solely on genetic merit. High levels of relatedness can reduce genetic diversity, increase the probability of deleterious alleles becoming fixed, and ultimately lead to inbreeding depression [31]. Therefore, developing index selection methods that balance genetic gain and control genetic relatedness is of paramount importance [20, 31]. In particular, optimizing selection decisions by accounting for genomic relationships among individuals can prevent the excessive selection of closely related lines and preserve long-term genetic variability [20, 31].

In practice, many GS-based selection strategies are yet to incorporate constraints or penalties for genetic relatedness, primarily due to the complexity of modeling these aspects jointly. However, recent approaches have proposed modifications to the standard index framework to include penalties on coancestry or inbreeding coefficients, often through quadratic programming or optimization models [1, 20]. These methods aim to find a compromise between maximizing the overall index value and maintaining genetic diversity, which is essential for sustainable breeding programs [20, 31].

The importance of controlling genetic relatedness cannot be overstated. Populations with high levels of genetic relatedness may suffer from reduced vigor, fertility, and stress tolerance, undermining years of breeding progress. Moreover, maintaining genetic diversity is essential for long-term selection response, as it ensures that future generations have sufficient variability to respond to new selection pressures, such as emerging diseases or climate extremes. Therefore, any practical selection strategy must ensure that selection does not compromise genetic health and adaptability.

Considering this context, the present study introduces a new genomic multi-trait index selection approach that explicitly integrates control of genetic relatedness to enhance the identification of superior candidates for advancement within the breeding pipeline. The proposed method will integrate genomic data for multiple traits, weighted by their relative importance, and optimize the selection of individuals by maximizing index values while minimizing the genomic average pairwise relatedness or expected inbreeding of the selected set. The framework will be based on optimization theory, particularly binary quadratic programming, to allow for both flexibility and constraint satisfaction in realistic breeding scenarios.

This approach addresses a pressing need in plant breeding programs that seek to implement GS in a sustainable manner. By balancing genetic gain and the preservation of genetic diversity, the proposed index enables the identification of promising candidates for advancement in the breeding pipeline under a multi-trait context. The application of this method will be evaluated on real breeding datasets, where its performance will be compared against a linear programming multi-trait selection index (LPMSI). Ultimately, this research aims to contribute to the design of more robust and sustainable genomic selection pipelines in the current plant breeding.

## Materials and methods

### Data sets

In Table 1 we provide a description of the five data sets used in this research. The description focuses on type of crop, number of traits, number of markers and number of genotypes.

**Table 1:** Data set description

Dataset	Country	Crop	No. Traits	No. markers	No. genotypes	References
Wheat	Mexico	Wheat	4	1279	599	Crossa et al. [7]
Groundnut	India	Groundnut	4	58,000	318	Pandey et al. [24]
Indica,	Uruguay	Rice	4	92,430	327	Monteverde et al. [23]
Japonica	Uruguay	Rice	4	44,598	320	Monteverde et al. [23]
*Rice*	Philippines	Rice	6	73,147	369	Spindel et al. [27]

**Table 2:** Pearson´s correlation (lower triangular matrix) and significance (Upper triangular matrix) of the four traits of each data set

Dataset	Trait	T1	T2	T3	T4
Wheat	T1	1.000	0.629	< 0.001	0.003
Wheat	T2	− 0.020	1.000	< 0.001	< 0.001
Wheat	T3	− 0.193	0.661	1.000	< 0.001
Wheat	T4	− 0.123	0.411	0.388	1.000
Groundnut	Trait	NPP	PYPP	SYPP	YPH
Groundnut	NPP	1.000	0.000	0.000	0.000
Groundnut	PYPP	0.787	1.000	0.000	0.000
Groundnut	SYPP	0.821	0.965	1.000	0.000
Groundnut	YPH	0.718	0.869	0.840	1.000
Indica	Trait	GC	GY	PH	PHR
Indica	GC	1.000	0.000	0.346	0.001
Indica	GY	0.193	1.000	0.000	0.000
Indica	PH	− 0.052	− 0.329	1.000	0.408
Indica	PHR	− 0.189	0.210	− 0.046	1.000
Japonica	Trait	GC	GY	PH	PHR
Japonica	GC	1.000	0.000	0.007	0.000
Japonica	GY	0.372	1.000	0.000	0.000
Japonica	PH	0.149	− 0.247	1.000	0.341
Japonica	PHR	− 0.493	− 0.285	− 0.053	1.000
Rice	Trait	CulmL	Exs	FGP	Flg.LA
Rice	CulmL	1.000	0.652	0.457	0.000
Rice	Exs	− 0.025	1.000	0.693	0.001
Rice	FGP	0.042	− 0.022	1.000	0.940
Rice	Flg.LA	0.310	0.179	0.004	1.000

P-values < 0.05 are statically significant

It is important to note that the five datasets used in this study are publicly available and were obtained from the references listed for each dataset in Table 2. Each data set contains the best linear unbiased estimates (BLUEs) for each trait that were computed after taking the applied experimental designs into account. The genomic relationship matrix was computed using the marker data of each data set as proposed by Vanraden [32]. These datasets are accessible through the corresponding GitHub repository: https://github.com/osval78/QPMTSI.

## BLUEs preprocessing

### Step 1: standardization of each trait in each data set

Before combining traits into a single index, and due to the fact that each trait has a different scale, it is important to standardize each trait to ensure they are on the same scale and to control for differences in measurement units or variance. Standardization is particularly essential when traits have different units or variances [14, 25].

Standardization is commonly done by calculating y-scores:

$$\:{y}_{ki}=\frac{BLU{E}_{ji}-{\mu\:}_{j}}{{\sigma\:}_{j}}$$

where $\:BLU{E}_{ji}$ is the breeding value of trait $\:j$ for individual $\:i$;$\:{\:\mu\:}_{j}$ and $\:{\sigma\:}_{j}$ are the mean and standard deviation of trait $\:j$, respectively.

### Step 2: adjusting the direction of unfavorable traits

For traits where lower values are preferred (e.g., disease score, plant height if too tall is undesirable), the sign of the standardized BLUEs value should be reversed so that higher standardized scores always reflect more favorable breeding values. For a trait $\:j$ where lower is better:

$$\:{y}_{ji*}={-y}_{ji}$$

This transformation ensures that positive values consistently represent desirable performance, simplifying index construction and interpretation (Mulamba and Mock 1978).

### Step 3: minimum desired genetic gain for each trait ($\:{\boldsymbol{R}}_{\boldsymbol{j}}$)

The minimum desirable genetic gain ($\:{R}_{j})$ indicated for a given trait, which allows to assign different importance to each trait, is directly related to the “economic weight” (higher yield; or higher wheat quality traits implying higher bread quality, etc.). $\:{R}_{j}$ reflect the relative importance of improving trait $\:\mathrm{j}$, and are often derived from economic models, expert opinion, or genetic/phenotypic correlations with a desired trait [14]. In both methods evaluated in this research, that are described in the next section, the $\:{R}_{j}$ values are defined as follows:

$\:{R}_{j}$ under a base situation, the objective function defined by all traits is maximized, and the desired minimum gain for each trait is 0, that is, $\:{R}_{j}=0\%$ $\:\forall\:j$.

$\:{R}_{j}$ under specific situations, the objective function based on all traits is maximized, and the desired minimum gain for each trait is specified as a percentage between 0 and 100%.

### Quadratic programing Multi-trait index selection (QPMSI) method

The goal of this article is to propose a procedure for selecting the optimal number of individuals based on the estimated breeding values of the $\:n$ different candidates. This objective involves a binary quadratic programming optimization problem whose goal is to find the set of $\:s$ possible individuals that maximizes their combined group’s performance metric. This optimization consists of the search for the vector $\:\boldsymbol{x}$= *(*$\:{x}_{1}$,…, $\:{x}_{n}$*)* that maximizes the objective function Z as:

1 $$\begin{aligned}Z &=\sum\:_{i=1}^{n}\sum\:_{k=1}^{{p}_{0}}{y}_{ji}{x}_{i}-k\times\:\sum\:_{i=1}^{n}\sum\:_{j=1}^{n}{x}_{i}{G}_{ij}{x}_{j} \\ &=\sum\:_{i=1}^{n}\sum\:_{j=1}^{{p}_{0}}{y}_{ji}{x}_{i}-k\times\:{\boldsymbol{x}}^{T}\boldsymbol{G}\boldsymbol{x} \end{aligned}$$

where $\:{p}_{0}$ is the number of traits considered for maximization;$\:{\:y}_{ji}$ is the value of the standardized BLUEs of trait *j*, for individual *i*; $\:{G}_{ij}$ is the coordinate $\:ij$ of the genomic relationship matrix $\:\boldsymbol{G}$, that was computed using the scaled matrix of markers ($\:\boldsymbol{W})$ of order $\:n\times\:p,\:$using the method of VanRanden (2008) as $\:\boldsymbol{G}=\frac{\boldsymbol{W}{\boldsymbol{W}}^{T}}{p}$; $\:k$ by default is equal to one, that means that both the genetic gain and degree of relatedness have the same weights in the optimization process, however when $\:k>1$ the degree of relatedness have more weight and vice versa; $\:{x}_{i}$ is a binary decision variable with value 0 if individual $\:i$ is not selected and 1 if individual $\:i$ is selected, subject to the constraint:

2 $$\sum_{i=1}^{n}{x}_{i}=s$$

where $\:s$ is the number of individuals to be selected. For any given trait $\:j=\mathrm{1,2},\dots\:,{p}_{0}$ included in the objective function:

3 $$\sum_{i=1}^{n}{y}_{ji}{x}_{i}\ge\:{l}_{j},\:\mathrm{f}\mathrm{o}\mathrm{r}\:j=\mathrm{1,2},..,{p}_{0}$$

where $\:{p}_{0}$ is the number of traits; $\:{l}_{j}$ is the right-hand side value and is defined for trait *j* as:

4 $$\:{l}_{j}=\frac{{R}_{j}\times\:s}{100}$$

with $\:{R}_{j}\ge\:0\:$ being the minimum desirable genetic gain for trait *j*. In the proposed method, the weights for the different traits are defined by constraints and their definition can have several options according to the objectives. The decision variables are binary $\:{x}_{i}$ ∈ {0, 1}, and therefore, this is a binary quadratic programming problem. It is possible to use different traits in the objective function and in the constraints. The coefficients of the decision variables in the proposed objective function (Eq. 1) and the constraints (Eq. 2) are the genotypic effects BLUEs of each trait.

The objective function (1) subject to the constraints given in Eqs. (2–4) is maximized because it is designed to reward the selection of individuals that provide high overall genetic merit while penalizing the accumulation of genetic relatedness within the selected group. The first term of the objective function (1) represents the combined genetic merit of all selected individuals across all $\:{p}_{0}\:$traits, and by maximizing this term, the optimization procedure encourages the inclusion of individuals with superior breeding values. While the second term of the objective function (1) acts as a penalty for genetic relatedness between individuals. When two highly related individuals are selected (i.e., both $\:{x}_{i\:}$and $\:{x}_{j}\:$are 1), this term increases, reducing the objective function. Because the optimization seeks to maximize the function, the model naturally avoids selecting groups of individuals that are too closely related. Therefore, this structure of the proposed QPMSI guarantees that the optimal solution balances both goals: maximizing total genetic merit while minimizing overall relatedness. As a result, the selected individuals form a set of candidates that not only show high genetic potential but also preserve genetic diversity for future breeding cycles. The R library for implementing this method was the CVXR package [11]. Appendix A provides detailed instructions for installing and using the QPMSI R package, which we developed to implement both the LPMSI approach and the proposed QPMSI method.

### Linear programing multi-trait index selection (LPMSI) method

This method is similar to QPMSI, except that instead of using a quadratic objective function, it employs a linear objective function aimed at maximizing the following equation:

5 $$\:Z=\sum_{i=1}^{n}\sum_{j=1}^{{p}_{0}}{y}_{ji}{x}_{i}$$

This method was proposed by Surgy et al. [28] and also works with the same restriction given in Eqs. 2, 3 and 4. For this reason, the proposed QPMSI can be seen as a generalization of the LPMSI method proposed by Surgy et al. [28] to guarantee the selection of best individual with minimum genetic relatedness values. This method was implemented using lpSolve R package [4].

### Metrics for evaluation

The metrics used for evaluating the performance of the proposed method (QPMSI) compared to the LPMSI method were:

**Mean (M)**: M denotes the average genetic gain calculated for each method using the $\:s$ selected individuals, as follows.

$$\:Mea{n}_{QPMSI}=\frac{\sum\:_{i=1}^{n}\sum\:_{j=1}^{{p}_{0}}{y}_{ji}{x}_{i}}{s\times\:{p}_{0}} {\mathrm{and}}$$

$$\:Mea{n}_{LPMSI}=\frac{\sum\:_{i=1}^{n}\sum\:_{j=1}^{{p}_{0}}{y}_{ji}{x}_{i}}{s\times\:{p}_{0}}$$

Naturally, each metric was calculated using the subset of individuals ($\:{x}_{i}^{{\prime\:}}s$ values) selected by the respective method.

**Variance (V)**: To estimate this metric, the variance of the mean among of the $\:s$ selected individuals is first computed for each method, as follows: $\:{Var}_{QPMSI}={\boldsymbol{x}}^{T}\boldsymbol{G}\boldsymbol{x}/{s}^{2}$ and $\:{Var}_{LPMSI}={\boldsymbol{x}}^{T}\boldsymbol{G}\boldsymbol{x}/{s}^{2}$ respectively. As expected, each variance of the mean was derived based on the group of $\:s\:$individuals selected by the respective method.

**Mean_Var (MV)**: To compute this metric, we utilized the values obtained from the previously calculated metrics. Accordingly, the MV for each method was determined as follows.

$$\mathrm{MV}\_\mathrm{QPMSI} =\:{Mean}_{QPMSI}/\sqrt{{Var}_{QPMSI}}\,\, \mathrm{and} $$

$$\mathrm{MV}\_\mathrm{LPMSI}\:=\:\:{Mean}_{LPMSI}/\sqrt{{Var}_{LPMSI}}\:$$

This **MV** metric serves as a direct measure of the gain-to-degree of relatedness ratio, reflecting the efficiency of selection. A higher **MV** value indicates a greater expected genetic gain per unit of degree of relatedness, thereby representing more efficient selection relative to the associated increase in genetic relatedness.

**Mean_Relatedness (MR)**: To calculate this metric, we first extracted from the genomic relationship matrix $\:\boldsymbol{G}\:$the submatrix corresponding to the $\:s\:\:$genotypes selected by each method. From this submatrix, the upper triangular elements—excluding the diagonal and lower triangular elements—were isolated. The mean values of these elements were then used to estimate the average pairwise relatedness for each selection strategy ($\:{\mathrm{M}\mathrm{R}}_{QPMSI}$ and $\:{\mathrm{M}\mathrm{R}}_{LPMSI}$). The average degree of relatedness was computed based on the individuals selected by each respective method.

It is important to note that the **V** and **MR** metrics both quantify the degree of relatedness among the selected individuals; however, they are not numerically identical. The key difference lies in the elements of the genomic relationship matrix considered during their computation. Specifically, the **V** metric includes the diagonal, lower, and upper triangular elements of the submatrix corresponding to the $\:s$ selected genotypes. In other words, **V** incorporates all entries of the genomic relationship matrix for the selected individuals, including the diagonal terms that represent self-relatedness.

In contrast, the **MR** metric is calculated only from the upper triangular elements—excluding the diagonal and lower triangular elements—of the genomic relationship submatrix. Thus, MR captures the average pairwise relatedness among the selected individuals by considering only the off-diagonal upper-triangle elements of the sumatrix. As a result, both metrics move in parallel and represent the same underlying concept of genetic relatedness, but each summarizes it differently. **MR** reflects the average pairwise relatedness, while **V** summarizes the overall contribution of genomic relationships—including both self-relatedness and pairwise relatedness—precisely matching the components incorporated into the QPMSI penalty term.

To implement the proposed QPMSI, we evaluated three values of $\:k$ (0.5, 1.0, and 1.5). Including the LPMSI, this resulted in four selection methods, which were compared in terms of the four performance metrics described earlier. To enable a clearer and more interpretable comparison, we calculated the relative efficiency of each method with respect to the best-performing method within each dataset and across datasets. The relative efficiencies, expressed as percentages, were computed using the following formulas. For metric **M** was computed as:

6 $$\:R{E}_{\boldsymbol{M}}=\left(\frac{{\boldsymbol{M}}_{bm}}{{\boldsymbol{M}}_{q}}-1\right)\times\:100$$

Where $\:{\boldsymbol{M}}_{bm}$ denotes the **M** of the best model and $\:{\boldsymbol{M}}_{q}$ denotes the **M** of the remaining models. For the **V** metric was computed with:

7 $$\:R{E}_{\boldsymbol{V}}=\left(\frac{{\boldsymbol{V}}_{q}}{{\boldsymbol{V}}_{bm}}-1\right)\times\:100$$

Where $\:{\boldsymbol{V}}_{bm}\:$ denotes the $\:\boldsymbol{V}$ of the best model and $\:{\boldsymbol{V}}_{q}$ denotes the $\:\boldsymbol{V}$ of the remaining models. For **MV** we used the following expression:

8 $$\:R{E}_{\boldsymbol{M}\boldsymbol{V}}=\left(\frac{{\boldsymbol{M}\boldsymbol{V}}_{bm}}{{\boldsymbol{M}\boldsymbol{V}}_{q}}-1\right)\times\:100$$

Where $\:{\boldsymbol{M}\boldsymbol{V}}_{bm}$ denotes the **MV** of the best model and $\:{\boldsymbol{M}\boldsymbol{V}}_{q}$ denotes the **MV** of the remaining models. Finally for **MR** we used:

9 $$\:R{E}_{\boldsymbol{M}\boldsymbol{R}}=\left(\frac{{\boldsymbol{M}\boldsymbol{R}}_{q}}{{\boldsymbol{M}\boldsymbol{R}}_{bm}}-1\right)\times\:100$$

where$\:\:{\boldsymbol{M}\boldsymbol{R}}_{bm}$ denotes the $\:\boldsymbol{M}\boldsymbol{R}$ of the best model and$\:\:{\boldsymbol{M}\boldsymbol{R}}_{q}$ denotes the $\:MR$ of the remaining models.

## Results

The results are organized into two main sections. The first section explores the degree of phenotypic correlation among traits within each dataset. The second section compares the performance of our proposed method, QPMSI, with the LPMSI approach. This comparison is presented through separate subsections for each dataset, followed by an additional subsection that summarizes results across all datasets.

### Analysis of phenotypic correlation between traits for each data set

Table 2 shows the correlations of the BLUEs between traits in the wheat dataset. Five of the six correlations are statistically significant (*P* < 0.05); however, only the correlation between traits T1 and T2 exceed 0.5 in absolute value.

In the groundnut dataset (Table 2). Here the six correlations are statistically significant (*P* < 0.05) and all the correlation between traits exceeds 0.5 in absolute value. While in the indica data set (Table 2) four out of the six correlations are statistically significant (*P* < 0.05); and any of the correlation between traits exceeds 0.5 in absolute value.

In this Japonica five out of the six correlations between the BLUEs are statistically significant (*P* < 0.05); and any of the correlation between traits exceeds 0.5 in absolute value (Table 2). While in the rice data set Table 2 among the six correlations evaluated, two were statistically significant (*P* < 0.05). However, none of the correlations exceeded 0.5 in absolute value, indicating only moderate associations between traits.

### Comparison between the QPMSI and LPMSI

Next, for each dataset and across all datasets, a comparison is conducted between the two evaluated approaches, LPMSI and QPMSI.

#### Data set 1: wheat

**Fig. 1:**
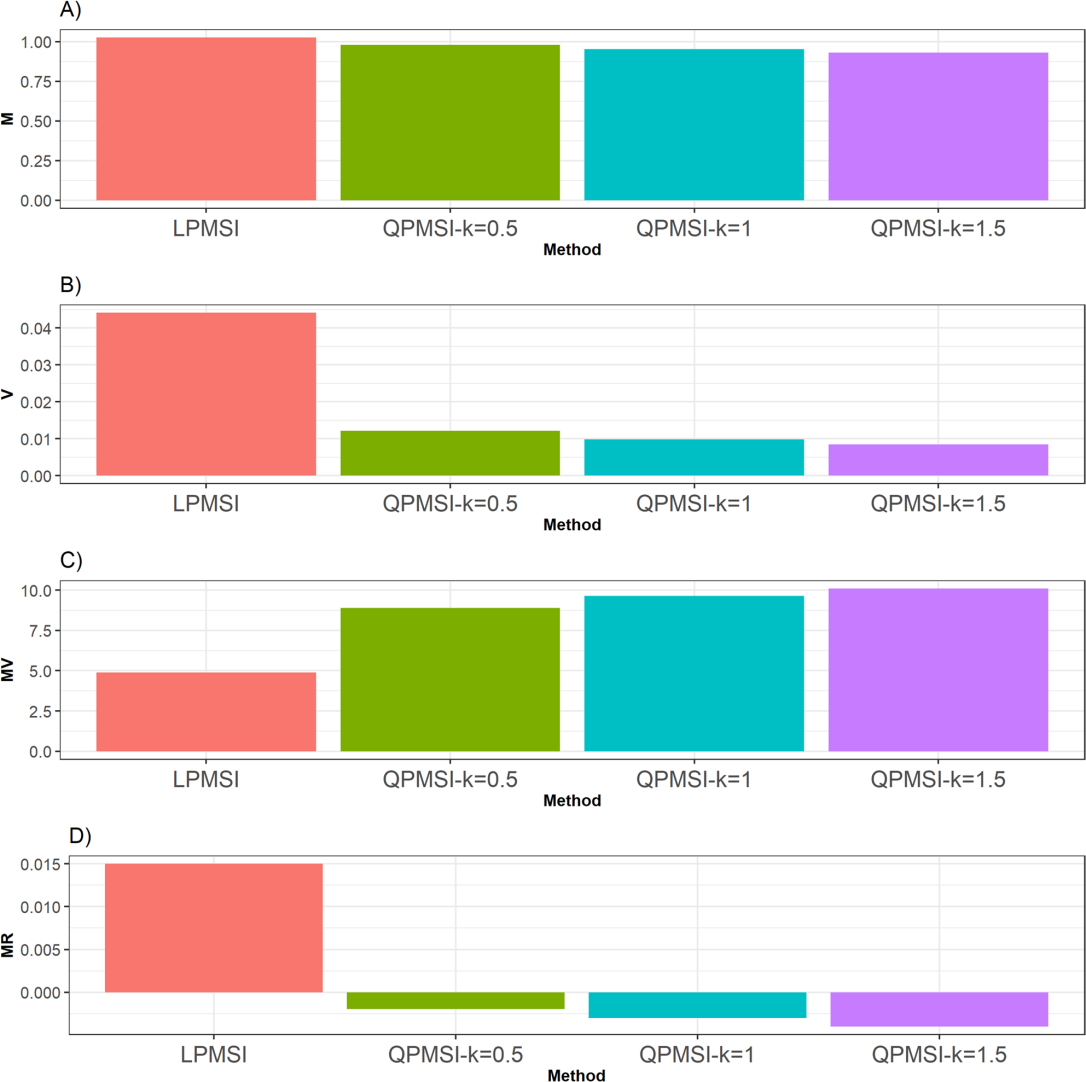
Selection performance for the Wheat data set. Selection performance was evaluated using four metrics: **A** Mean (**M**), which denotes the average genetic gain; **B** Variance (**V**), which measures the variability of the mean among the selected individuals; **C** Mean-Variance (**MV**), which computes the gain-to-degree of relatedness ratio; and **D** Mean Relatedness (**MR**), which reflects the average degree of relatedness among the selected individuals

For this dataset, as observed in Fig. 1, in terms of the **M** criterion, the LPMSI method outperformed the three versions of QPMSI by 4.78% (k = 0.5), 7.77% (k = 1), and 10.34% (k = 1.5). In contrast, for the **V** metric, all three versions of QPMSI outperformed LPMSI, with QPMSI at k = 1.5 achieved the best performance. Specifically, it surpassed QPMSI (k = 0.5) by 43.63%, QPMSI (k = 1) by 15.34%, and LPMSI by 420.66%. This observed large percentage difference for **V** (e.g., 420.65% for Wheat) is due to a change in the scale of the denominator (a quantity close to zero).

Similarly, for the **MV** metric, all QPMSI variants outperformed LPMSI with QPMSI (k = 1.5) provided the best performance improving QPMSI (k = 0.5) by 13.78%, QPMSI (k = 1) by 4.89%, and LPMSI by 106.79% (Fig. 1).

Finally, with the **MR** metric, the three QPMSI methods were consistently superior to LPMSI with QPMSI (k = 0.5) yielded the highest score outperforming others including QPMSI (k = 1) by 52.14%, QPMSI (k = 1.5) by 102.86%, and LPMSI by 660.71%. Details can be found in Table 3.

**Table 3:** Comparison of the three QPMSI versions (k = 0.5, 1, and 1.5) with the LPMSI across four metrics—Mean (M), variance (V), Mean-Variance (MV), and Mean-Relatedness (MR)—in the five evaluated datasets and across data sets

Dataset	k	Method	M	V	MV	MR
Wheat	0.5	QPMSI	0.980	0.012	8.886	− 0.002
Wheat	1	QPMSI	0.953	0.010	9.641	− 0.003
Wheat	1.5	QPMSI	0.931	0.008	10.113	− 0.004
Wheat		LPMSI	1.027	0.044	4.890	0.015
Groundnut	0.5	QPMSI	1.542	1.537	1.243	0.764
Groundnut	1	QPMSI	1.383	1.511	1.125	0.751
Groundnut	1.5	QPMSI	1.295	1.503	1.056	0.747
Groundnut		LPMSI	7.331	1.833	1.771	1.377
Indica	0.5	QPMSI	0.718	0.015	5.855	− 0.008
Indica	1	QPMSI	0.701	0.012	6.286	− 0.008
Indica	1.5	QPMSI	0.702	0.012	6.288	− 0.008
Indica		LPMSI	0.756	0.064	2.992	0.015
Japonica	0.5	QPMSI	0.730	0.020	5.112	− 0.005
Japonica	1	QPMSI	0.708	0.016	5.513	− 0.007
Japonica	1.5	QPMSI	0.687	0.014	5.707	− 0.008
Japonica		LPMSI	0.777	0.058	3.215	0.015
Rice	0.5	QPMSI	1.052	0.039	5.347	0.001
Rice	1	QPMSI	0.998	0.031	5.696	− 0.003
Rice	1.5	QPMSI	NA	NA	NA	NA
Rice		LPMSI	1.080	0.052	4.716	0.006
AcrossData	0.5	QPMSI	1.004	0.325	5.289	0.150
AcrossData	1	QPMSI	0.949	0.316	5.652	0.146
AcrossData	1.5	QPMSI	0.903	0.384	5.791	0.182
AcrossData		LPMSI	1.094	0.398	3.438	0.186

#### Data set 2: groundnut

**Fig. 2:**
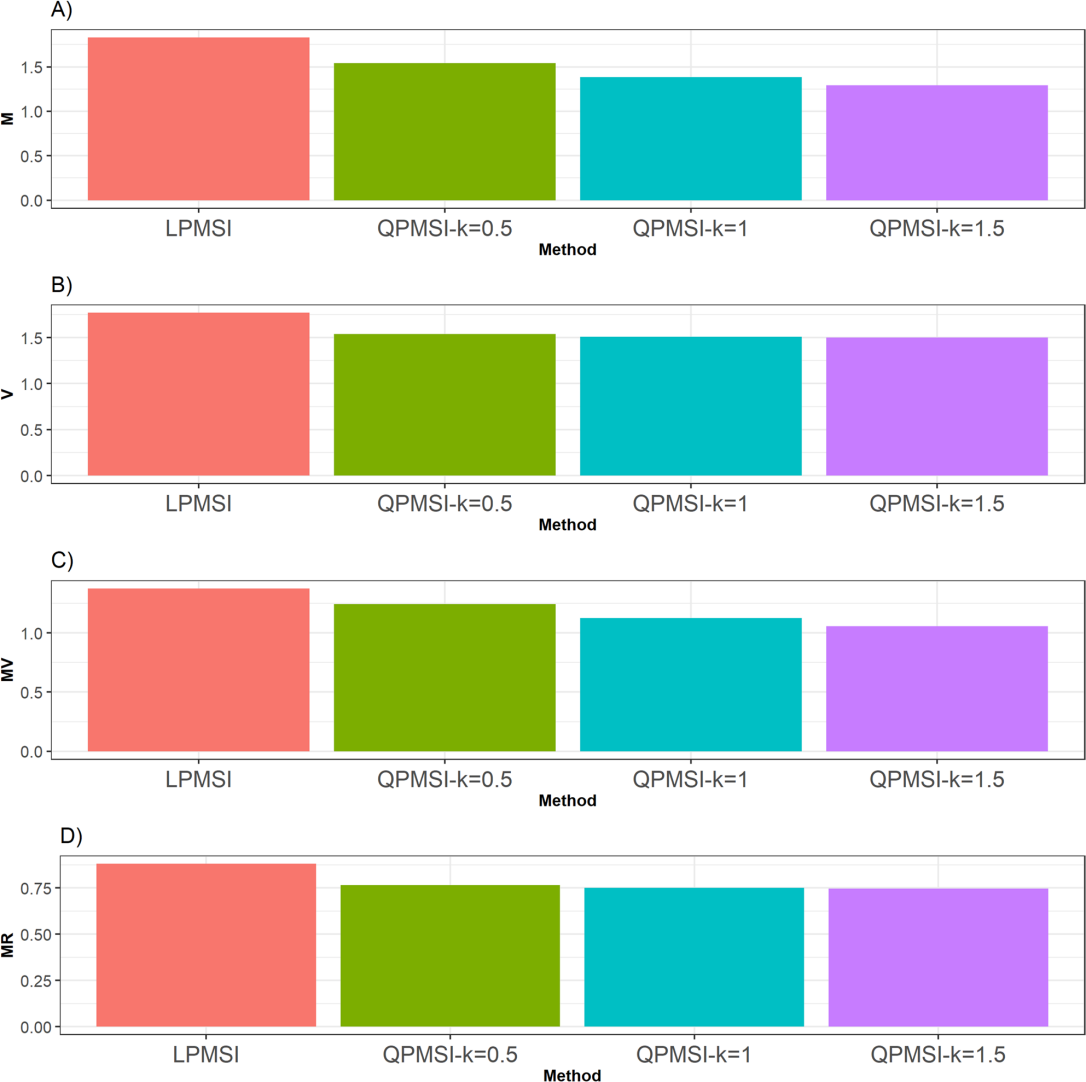
Selection performance for the Groundnut data set. Selection performance was evaluated using four metrics: (**A**) Mean (**M**), which denotes the average genetic gain; (**B**) Variance (**V**), which measures the variability of the mean among the selected individuals; (**C**) Mean-Variance (**MV**), which computes the gain-to-degree of relatedness ratio; and (**D**) Mean Relatedness (**MR**), which reflects the average degree of relatedness among the selected individuals

In this dataset, with the **M** criterion, the LPMSI method yielded the highest performance (Fig. 2), surpassing the three variants of QPMSI by 18.87% (at k = 0.5), 32.52% (at k = 1), and 41.58% (at k = 1.5). Conversely, under the **V** metric, all QPMSI variants outperformed LPMSI, with QPMSI (k = 1.5) demonstrating the strongest performance (Fig. 2). In particular, it exceeded QPMSI (k = 0.5) by 2.32%, QPM3I (k = 1) by 0.56%, and LPMSI by 17.86%.

Regarding the **MV** metric, the LPMTSI method outperformed all QPMSI variants, improving QPMSI (k = 0.5) by 10.76%, QPMSI (k = 1) by 22.41%, and QPMSI (k = 1.5) by 30.41% (Fig. 2). Finally, for the **MR** metric, the QPMSI methods consistently outperformed LPMSI (Fig. 2), with QPMSI (k = 1.5) emerging as the top performer. Specifically, it surpassed QPMSI (k = 0.5) by 2.34%, QPMSI (k = 1) by 0.60%, and LPMSI by 17.95% (Table 3).

#### Data set 3: indica

**Fig. 3:**
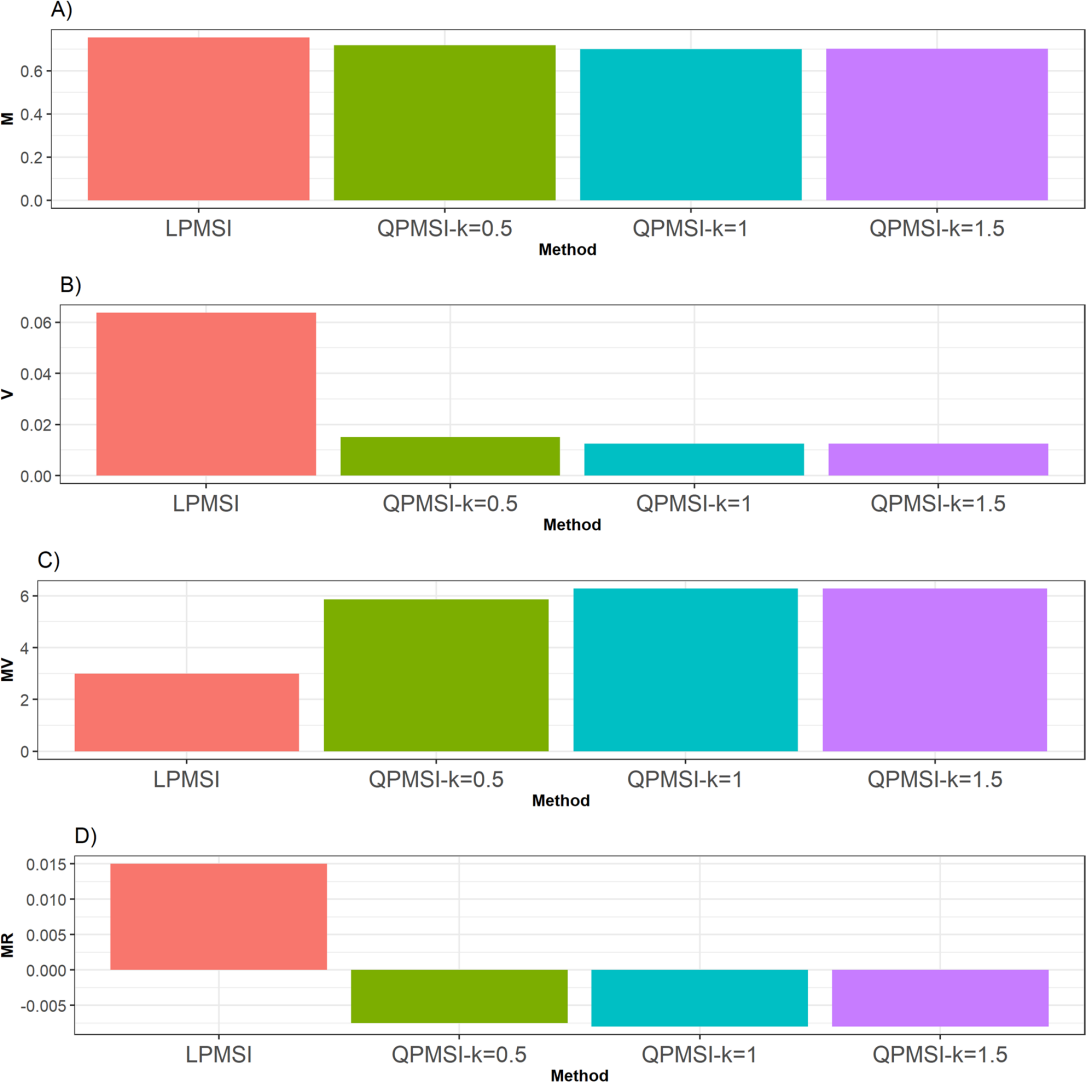
Selection performance for the Indica data set. Selection performance was evaluated using four metrics: (**A**) Mean (**M**), which denotes the average genetic gain; (**B**) Variance (**V**), which measures the variability of the mean among the selected individuals; (**C**) Mean-Variance (**MV**), which computes the gain-to-degree of relatedness ratio; and (**D**) Mean Relatedness (**MR**), which reflects the average degree of relatedness among the selected individuals

For this dataset, in relation to the **M** criterion, the LPMSI method achieved the best performance exceeding the three QPMSI variants by 5.19% (at k = 0.5), 7.74% (at k = 1), and 7.7% (at k = 1.5) (Fig. 3). In contrast, under the **V** metric, all QPMSI variants outperformed LPMSI, with QPMSI (k = 1 and k = 1.5) delivered the highest score outperforming QPMSI (k = 0.5) by 20.92% and LPMSI by 412.23% (Fig. 3).

For the **MV** metric, every QPMSI variant surpassed LPMSI with QPMSI (k = 1 and k = 1.5) showing the best performance (Fig. 3). These variants improved the QPMSI (k = 0.5) by 7.40% and LPMSI by 110.10%.

Finally, regarding the **MR** metric, the QPMSI methods consistently demonstrated superiority over LPMSI (Fig. 3), with QPMSI (k = 0.5) standing out as the best performer. In particular, it surpassed QPMSI (k = 1and k = 1.5) by 6.67% and LPMSI by 100%. Details can be found in Table 3.

#### Data set 4: japonica

**Fig. 4:**
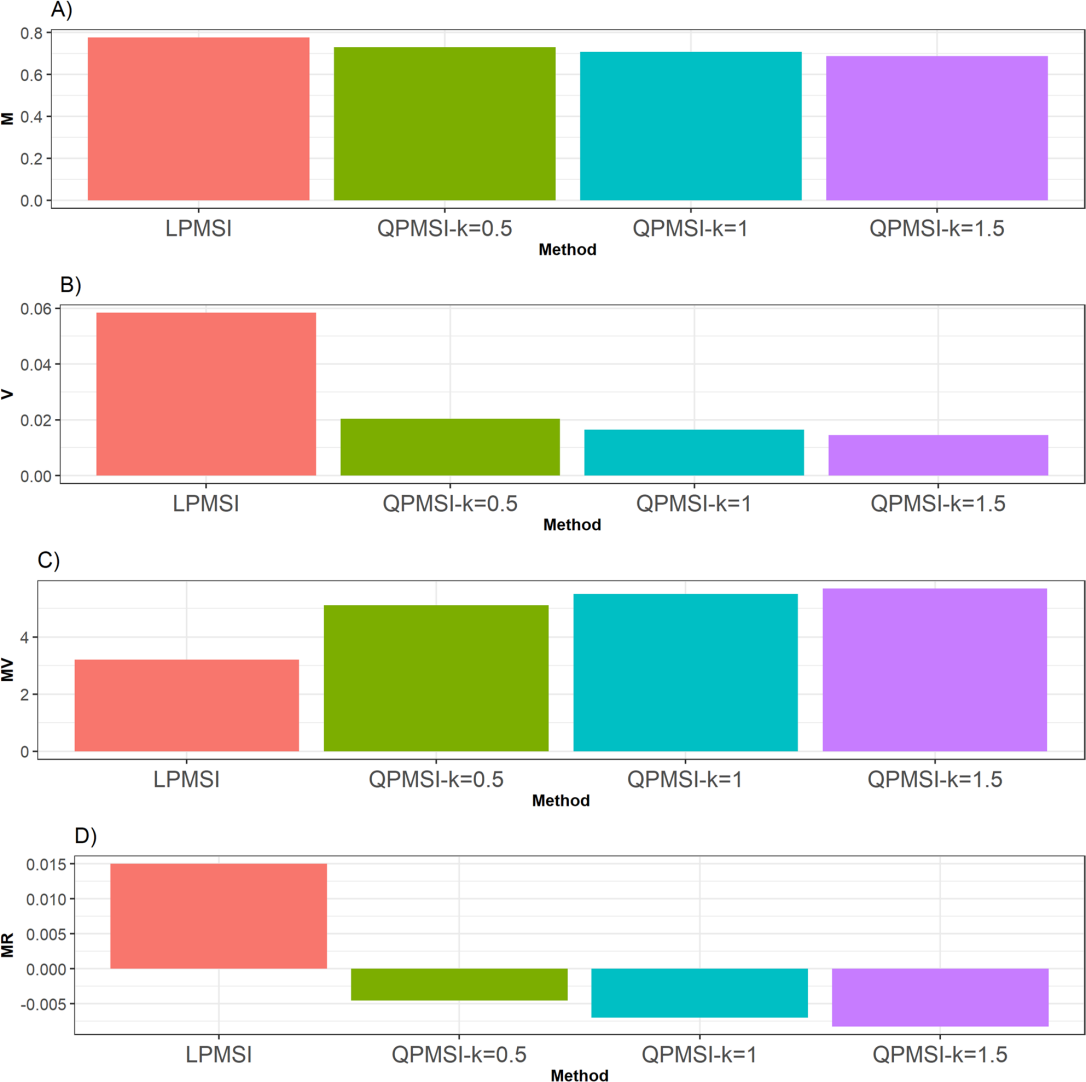
Selection performance for the Japonica data set. Selection performance was evaluated using four metrics: (**A**) Mean (**M**), which denotes the average genetic gain; (**B**) Variance (**V**), which measures the variability of the mean among the selected individuals; (**C**) Mean-Variance (**MV**), which computes the gain-to-degree of relatedness ratio; and (**D**) Mean Relatedness (**MR**), which reflects the average degree of relatedness among the selected individuals

For this dataset, Fig. 4 illustrates that, according to the **M** criterion, the LPMSI method achieved the highest performance, surpassing the three QPMSI variants by 6.48% (for *k* = 0.5), 9.82% (for *k* = 1), and 13.07% (for *k* = 1.5). In contrast, when evaluated using the **V** metric, all QPMSI approaches outperformed LPMSI, with QPMSI (k = 1.5) delivering the best overall result. Specifically, it exceeded QPMSI (k = 0.5) by 40.58%, QPMSI (k = 1) by 13.62%, and LPMSI by a remarkable 302.93% (see Fig. 4).

A similar pattern was observed for the **MV** metric, where all QPMSI variants outperformed LPMSI (Fig. 4). Among them, QPMSI (k = 1.5) exhibited the strongest performance, surpassing QPMSI (k = 0.5) by 11.66%, QPMSI (k = 1) by 3.53%, and LPMSI by 77.53%. Finally, regarding the **MR** metric, the QPMSI methods again consistently outperformed LPMSI (Fig. 4), with QPMSI (k = 0.5) emerging as the top performer. Specifically, it outperformed QPMSI (k = 1) by 54.84%, QPMSI (k = 1.5) by 82.84%, and LPMSI by 231.79%. Additional numerical details are provided in Table 3.

#### Data set 5: rice

**Fig. 5:**
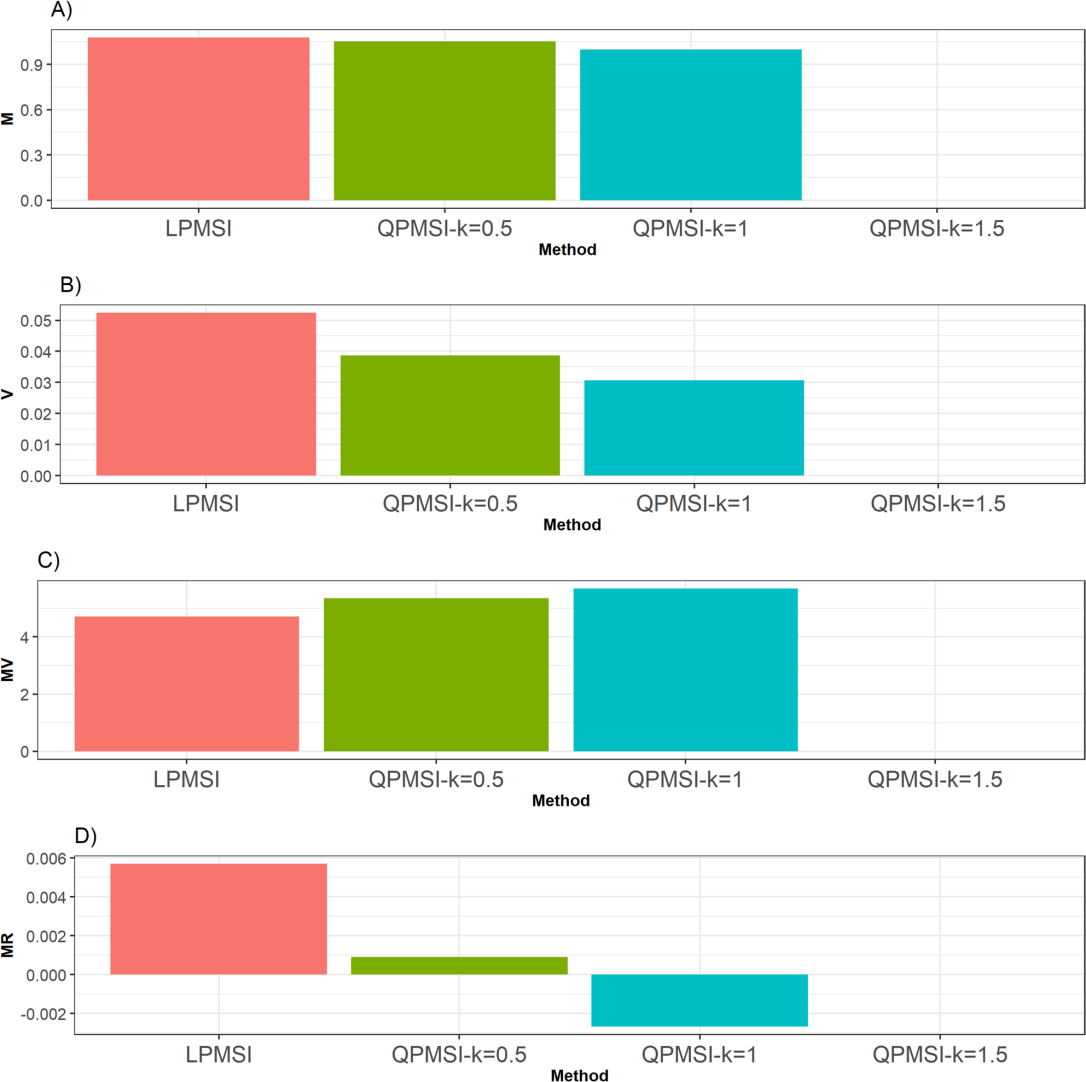
Selection performance for the Rice data set. Selection performance was evaluated using four metrics: (**A**) Mean (**M**), which denotes the average genetic gain; (**B**) Variance (**V**), which measures the variability of the mean among the selected individuals; (**C**) Mean-Variance (**MV**), which computes the gain-to-degree of relatedness ratio; and (**D**) Mean Relatedness (**MR**), which reflects the average degree of relatedness among the selected individuals

Under the **M** criterion, the LPMSI method achieved the highest performance (Fig. 5), surpassing the two QPMSI variants by 2.66% (at *k* = 0.5) and 8.17% (at *k* = 1). In contrast, according to the **V** metric, both QPMSI approaches outperformed LPMSI, with QPMSI (k = 1) delivering the best performance. Specifically, it exceeded QPMSI (k = 0.5) by 25.97% and LPMSI by 70.67% (Fig. 5).

A similar pattern was observed for the **MV** metric, where both QPMSI variants again outperformed LPMSI, with QPMSI (k = 1) demonstrating the strongest results (Fig. 5). This version improved upon QPMSI (k = 0.5) by 6.52% and upon LPMSI by 20.78%.

Finally, for the **MR** metric, the QPMSI methods consistently outperformed LPMSI (Fig. 5), with QPMSI (k = 0.5) emerging as the top performer. It exceeded QPMSI (k = 1) by 194.58% and LPMSI by 528.21%. Detailed numerical results are provided in Table 3.

#### Summary across data sets

In terms of the **M** criterion, we observed that across the five datasets, the LPMSI method consistently achieved the best performance surpassing the QPMSI by 8.97% (k = 0.5), 15.37% (k = 1) and 21.1% (k = 135.5) across datasets. This outcome was expected, as LPMSI does not impose constraints on the degree of relatedness, which allowed it to deliver the highest average genetic gain. However, this gain came at the cost of selecting lines with a high degree of relatedness.

Regarding the **V** criterion, it was evident that in all five datasets, every QPMSI variant surpassed the performance of LPMSI by 22.54% (when k = 0.5), 25.9% (when k = 1.0) and 3.5% (when k = 1.5) across datasets. Such a result was anticipated, as QPMSI incorporates restrictions on the degree of relatedness among lines and for this reason the QPMSI methods had lower variance. Nevertheless, these restrictions led to the selection of individuals with, on average, lower genetic gain compared to those chosen by the LPMSI method.

With respect to the **MV** criterion, the QPMSI variants outperformed LPMSI in four of the five datasets and the observed gains across datasets were of 53.82% (when k = 0.5), 64.4% (when k = 1) and of 68.44% when k = 1.5. This outcome was anticipated, as the variance of QPMSI is reduced due to the constraints imposed on the degree of relatedness among lines. Nonetheless, these constraints also resulted in the selection of individuals that, on average, exhibited lower genetic gain than those selected by the LPMSI method.

Finally, regarding the **MR** criterion, it was clear that across all five datasets, each QPMSI variant outperformed LPMSI. The improvements observed across the datasets reached 24.01% when (k = 0.5), 27.51% when (k = 1), and 2.58% when (k = 1.5). This finding was expected, since QPMSI applies constraints designed to prioritize the selection of individuals with minimal levels of inbreeding. However, these constraints also resulted in the selection of individuals that, on average, achieved lower genetic gain compared with those identified by the LPMSI method.

**Fig. 6:**
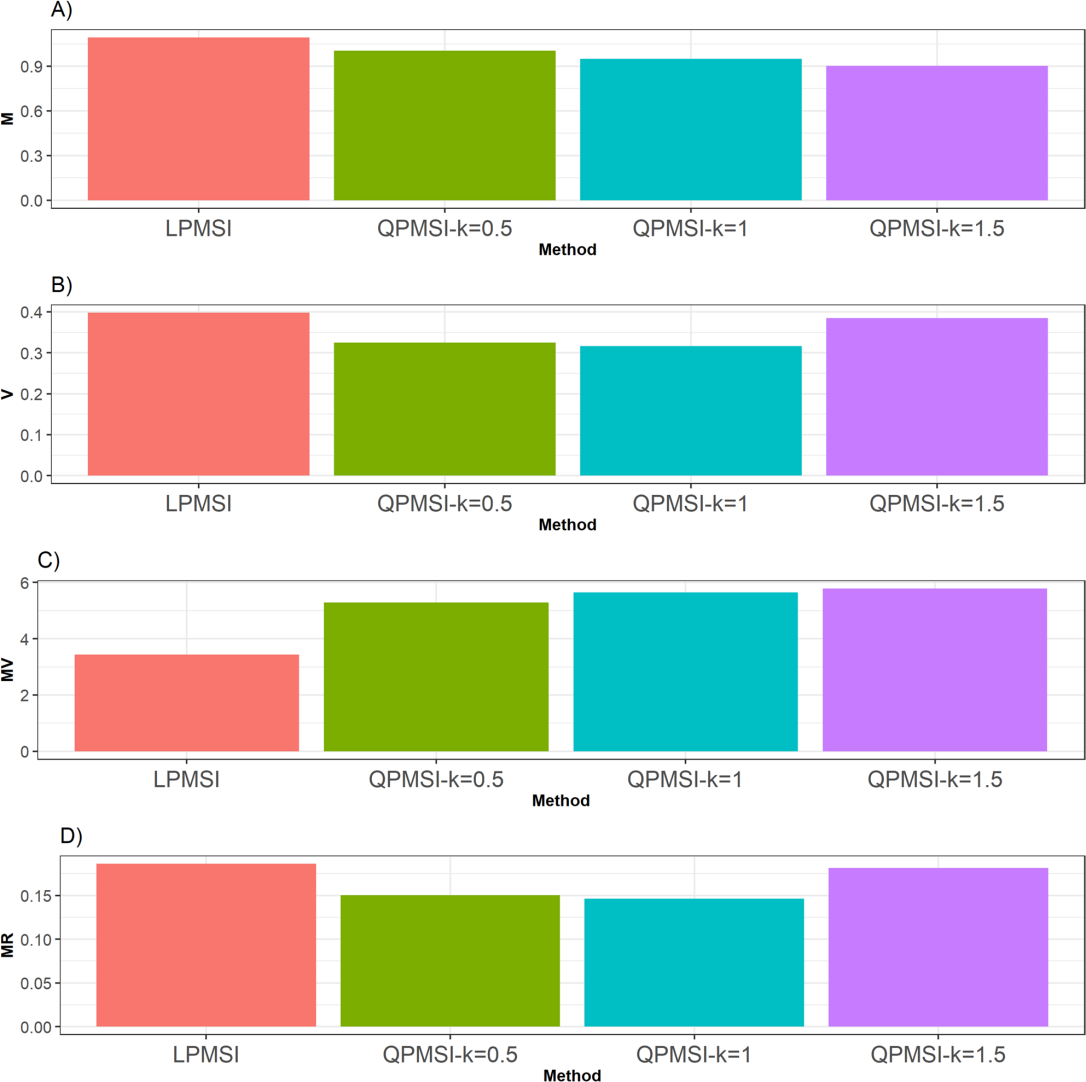
Selection performance across datasets. Selection performance was evaluated using four metrics: (**A**) Mean (**M**), which denotes the average genetic gain; (**B**) Variance (**V**), which measures the variability of the mean among the selected individuals; (**C**) Mean-Variance (**MV**), which computes the gain-to-degree of relatedness ratio; and (**D**) Mean Relatedness (**MR**), which reflects the average degree of relatedness among the selected individuals

## Discussions

The results of this study underscore the critical importance of integrating optimization-based frameworks into GS, particularly for breeding programs dealing with multi-traits. While GS has emerged as a transformative approach in both plant and animal breeding by enabling early and accurate prediction of breeding values [2, 15, 22], most implementations have traditionally focused on single-trait selection or ignored the negative impact of long-term inbreeding. However, sustainable genetic improvement requires a balance between short-term genetic gain and long-term genetic diversity [31].

It is important to emphasize that the proposed QPMSI framework is specifically designed to identify superior candidates for advancement within the breeding pipeline under a multi-trait scenario, while simultaneously targeting the selection of individuals with high genetic merit and low degrees of relatedness. In contrast, the established Optimal Contribution Selection (OCS) by Meuwissen et al. [20] approach is primarily designed for choosing parents for the subsequent generation and assigning their respective contributions to the crossing scheme, typically within a univariate context with the same dual objective of maximizing merit and minimizing relatedness. Therefore, to ensure methodological alignment and a meaningful comparison, the proposed method was evaluated exclusively against the LPMSI.

Our proposed QPMSI method addresses this challenge by employing a Binary Quadratic Programming (BQP) framework to identify individuals that jointly optimize a multi-trait selection index while minimizing genetic relatedness. The initial multiple-trait selection indices proposed by Hazel [14] have been extended through the integration of modern genomic information and computational optimization techniques. Unlike linear programming approaches, which may oversimplify the complex interactions among individuals and traits, quadratic programming enables the direct modeling of coancestry via the genomic relationship matrix [32], thereby allowing the simultaneous optimization of genetic gain and genetic diversity.

A key advantage of QPMSI is its capacity to manage genetic diversity through the explicit penalization of genetic relatedness among selected individuals. Traditional truncation selection and index-based approaches often produce rapid increases in coancestry, particularly under high selection intensity or when multiple traits are genetically correlated [26]. In contrast, QPMSI incorporates a constraint on pairwise relatedness that directly mitigates this issue, promoting a more diverse and genetically balanced set of selected candidates.

The empirical results support this rationale. When evaluated using the MV criterion, QPMSI outperformed LPMSI in four of the five genomic datasets analyzed. The observed gains reached 53.823% when (k = 0.5), 64.398% for (k = 1), and 68.437% for (k = 1.5), confirming that integrating genetic relatedness control improves index performance. By limiting the relatedness of selected individuals, QPMSI effectively reduces the variance of the selection index and ensures a more stable distribution of genetic contributions. However, this constraint also introduces a trade-off: although QPMSI enhances the MV measure, the average genetic gain of selected individuals is often slightly lower than that observed with LPMSI. This outcome reflects the fundamental balance in breeding programs between maximizing short-term genetic response and maintaining long-term genetic diversity.

The consistency of these findings across five diverse datasets further highlights the robustness and applicability of the proposed method. In each case, QPMSI achieved lower average pairwise relatedness among the selected individuals compared with LPMSI, with the strongest differences observed in the MV criterion. Collectively, these results show that QPMSI not only improves selection outcomes but also provides a methodologically sound and sustainable strategy adaptable to a range of genomic architectures and breeding contexts.

These findings are in line with previous studies showing that quadratic programming-based approaches can more effectively manage genetic diversity [16, 18]. Also, by specifying a value of $\:k$ different to one we can perform the optimization process with more weight to maximize the genetic gain (when $\:k<1)$ or to minimizing the inbreeding level ($\:k>1$).

Because the parameter $\:k$ determines the relative emphasis placed on maximizing genetic gain versus minimizing genetic relatedness, it functions as a tuning mechanism that enables breeders to manage the trade-off between these two competing objectives. A value of ($\:k$ = 1) generally provides a balanced compromise; however, larger values of (k) may be required when stronger penalties on relatedness are desired, such as targeting negative or near-zero MR values. For this reason, we recommend ($\:k$ = 1) as a reasonable default.

The role of $\:k$ is conceptually similar to specifying a maximum acceptable inbreeding rate in OCS, where breeders explicitly regulate the contributions of related individuals to maintain long-term genetic diversity [20, 26]. By evaluating multiple values of $\:k$, breeders can empirically characterize the gain–relatedness frontier for their population and select a configuration that satisfies both short-term selection goals and long-term sustainability [12].

However, as noted by one reviewer, if $\:k$ is set too large, the optimization problem may no longer have a feasible solution. Therefore, we suggest using (k = 1) as a starting point, and if additional emphasis on reducing relatedness is desired, gradually increasing (k) to ensure that feasible solutions are still obtained for values greater than 1.

Furthermore, our framework accounts for trait-specific economic weights, allowing breeders to prioritize traits based on their breeding goals. This is particularly important in modern breeding programs where trade-offs among traits (e.g., yield versus disease resistance) are common and where economic or environmental constraints must be considered [9]. By integrating these weights directly into the optimization objective, QPMSI ensures that selection decisions are aligned with long-term breeding objectives.

From a computational perspective, the binary quadratic programming formulation—although more complex than linear alternatives—has become increasingly tractable thanks to advances in solver technology and computing power [13]. To facilitate its implementation, Appendix A describes the procedures for installing and using the QPMSI R package, which we developed to support both the LPMSI approach and the proposed QPMSI method. These developments make QPMSI a practical tool for large-scale breeding programs that manage high-dimensional genomic and phenotypic data. Furthermore, the QPMSI R package offers a user-friendly and accessible platform for implementing both LPMSI and QPMSI in applied breeding contexts.

However, as demonstrated in our empirical findings, the proposed QPMSI method is specifically designed to select individuals with high genetic gain and exhibiting minimal inbreeding, which naturally results in some reduction in average genetic gain relative to LPMSI. Unlike LPMSI—which solely maximizes genetic gain—QPMSI employs a dual-objective optimization strategy that simultaneously enhances genetic improvement and mitigates genetic relatedness.

This integrated approach offers several substantial advantages. First, by constraining genetic relatedness, QPMSI helps to preserve genetic diversity over successive breeding cycles, thereby reducing the risk of genetic bottlenecks, loss of adaptive variation, and the accumulation of deleterious alleles. These benefits align with recognized breeding principles on controlling coancestry to sustain long-term gain [20]—a strategy akin to OCS, which balances selection response with genetic conservation under a uni-trait framework [20]—ensuring stable performance across generations. In fact, methods that incorporate coancestry constraints have been shown to yield 21–60% greater genetic gains at equivalent rates of inbreeding compared to traditional BLUP-EBV selection [21]—highlighting the efficacy of such dual-objective methods.

Second, QPMSI’s constraint on genetic relatedess makes it particularly applicable in real-world breeding programs with limited germplasm or small effective population sizes, where unchecked relatedness could undermine future selection gains. By maintaining heterozygosity and effective population size, QPMSI ensures that selection response remains sustainable and resilient to environmental changes [6, 30].

Moreover, this balanced strategy supports long-term breeding objectives, such as adaptation across diverse environments, disease resistance, or resilience to climate change. While it may compromise short-term gain slightly, QPMSI enhances long-run sustainability, genomic integrity, and breeding program robustness.

Importantly, this study contributes to the growing body of literature emphasizing OCS strategies in genomic selection [31]. While our method does not explicitly implement OCS, its formulation similarly balances the trade-off between genetic gain and diversity, aligning with the goals of OCS in practical breeding contexts.

It is important to emphasize that both methods (LPMSI and QPMSI), since they were specifically designed for multi-trait selection, allow the assignment of weights that specify the desired minimum genetic gain for each trait, expressed as a percentage value between 0 and 100%. This flexibility enables breeders to prioritize traits according to their relevance, ensuring that some traits contribute more significantly to the selection of the best individuals.

The ability to adjust these weights is of paramount importance because breeding objectives rarely treat all traits as equally valuable. In many cases, certain traits have a stronger economic impact, such as yield, disease resistance, or grain quality, while others may hold greater genetic importance for long-term population improvement, such as drought tolerance or root architecture. By assigning higher weights to these key traits, breeders can strategically direct selection towards outcomes that maximize both immediate and future benefits.

Moreover, this weighting system provides a practical mechanism to align breeding strategies with market demands, sustainability goals, and resource efficiency. For example, if a breeding program operates in an environment where water scarcity is a critical challenge, assigning a higher weight to drought tolerance ensures that the selected individuals not only perform well overall but also address this specific constraint. Similarly, in markets where consumers demand higher nutritional quality, greater weight can be given to protein or micronutrient content.

In this sense, the capacity to specify minimum genetic gains through weights is not merely a technical advantage but a strategic tool. It empowers breeders to balance trade-offs among traits, avoid the dilution of progress in economically vital traits, and ensure that the genetic improvement process aligns closely with both present needs and long-term breeding goals.

It is also important to note that, as with many optimization algorithms, the proposed QPMSI does not always guarantee a feasible solution. For instance, in the rice dataset, QPMSI failed to identify a feasible solution when k = 1.5. This limitation arises because quadratic programming with multiple constraints can become infeasible when the feasible region defined by the constraints shrinks or when conflicting objectives prevent convergence. Such failures are more likely as the algorithm’s complexity increases and as the number of traits under optimization grows, since each additional trait introduces new constraints and potential trade-offs. Consequently, the search space becomes more restricted, and the probability of incompatibility among constraints rises, making it harder for the algorithm to converge to a solution that satisfies all requirements simultaneously. For this reason, we suggest applying QPMSI with different values of k. In cases where the algorithm fails to converge, the LPMSI should be used as an alternative.

In summary, the QPMSI framework offers a robust, scalable, and flexible method for multi-trait genomic selection under inbreeding constraints. It extends the LPMSI theory into the genomic era by combining estimated breeding values, genetic relatedness information, and economic weighting in a unified optimization model. The approach represents a valuable advancement for breeders seeking to optimize selection decisions under increasingly complex breeding scenarios.

## Conclusions

In this study, we present a binary quadratic programming framework designed to select optimal candidate individuals in a multi-trait context, while simultaneously minimizing genetic relatedness. Using five real-world breeding datasets, our proposed method (QPMSI) demonstrated consistent superiority over the LPMSI approach by selecting individuals with high breeding values while simultaneously maintaining lower levels of genetic relatedness. This balance is reflected in substantial gains in the MV metric (gain-to-degree of relatedness ratio), with improvements of at least 53.823% across datasets. These results highlight the ability of QPMSI to deliver stronger short-term selection response without compromising long-term population diversity, addressing a long-standing challenge in breeding optimization methods. Notably, the method provides a practical and scalable framework that enables breeders to explore the trade-off between gain and relatedness in a controlled and data-driven manner. For this reason, it achieved superior control over inbreeding compared to the LPMSI developed by Surgy et al. [28]. This approach of balancing genetic gain and inbreeding control is crucial for sustaining long-term improvement across breeding cycles. This contribution is intended to lower technical barriers for breeders and researchers aiming to incorporate advanced optimization techniques into their selection strategies. We strongly encourage the adoption of this framework as a means to achieve enduring genetic progress while preserving genetic diversity and mitigating inbreeding—two fundamental objectives in contemporary breeding programs.

## Appendix A

**Script for installing and using QPMSI R package**

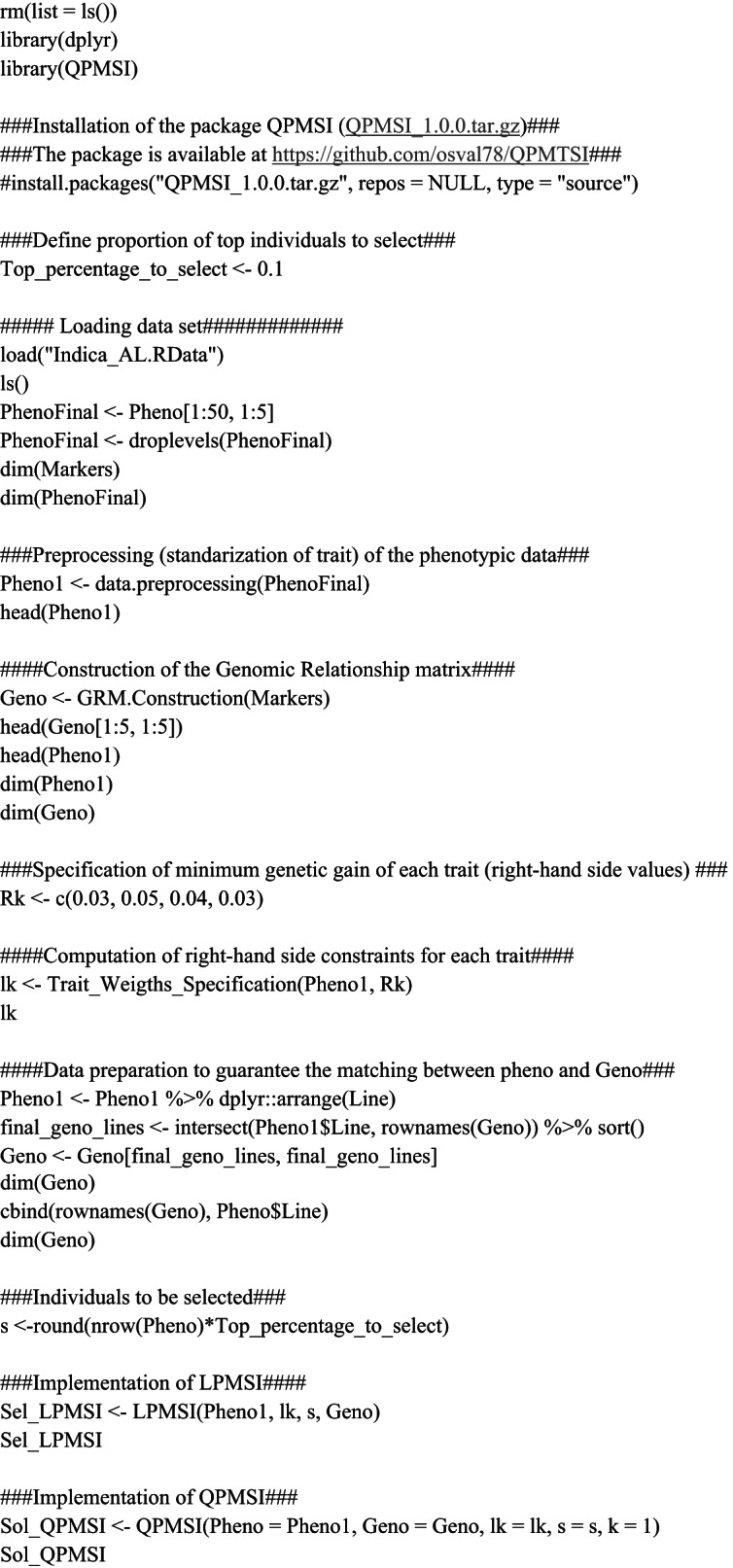



## Data Availability

Publicly available datasets were analyzed in this study. These datasets are accessible through the corresponding GitHub repository. https://github.com/osval78/QPMTSI.
